# Longitudinal assessment of reflexive and volitional saccades in Niemann-Pick Type C disease during treatment with miglustat

**DOI:** 10.1186/s13023-015-0377-8

**Published:** 2015-12-21

**Authors:** Larry A. Abel, Mark Walterfang, Matthew J. Stainer, Elizabeth A. Bowman, Dennis Velakoulis

**Affiliations:** Department of Optometry and Vision Sciences, The University of Melbourne, Melbourne, Australia; Melbourne Neuropsychiatry Centre, University of Melbourne, Melbourne, Australia; Neuropsychiatry Unit, Royal Melbourne Hospital, Melbourne, Australia; Active Vision Lab, School of Psychology, The University of Aberdeen, Aberdeen, Scotland

**Keywords:** Niemann-Pick type C, Miglustat, Saccades, Gain, Velocity, Antisaccade

## Abstract

**Background:**

Niemann-Pick Type C disease (NPC), is an autosomal recessive neurovisceral disorder of lipid metabolism. One characteristic feature of NPC is a vertical supranuclear gaze palsy particularly affecting saccades. However, horizontal saccades are also impaired and as a consequence a parameter related to horizontal peak saccadic velocity was used as an outcome measure in the clinical trial of miglustat, the first drug approved in several jurisdictions for the treatment of NPC. As NPC-related neuropathology is widespread in the brain we examined a wider range of horizontal saccade parameters and to determine whether these showed treatment-related improvement and, if so, if this was maintained over time.

**Methods:**

Nine adult NPC patients participated in the study; 8 were treated with miglustat for periods between 33 and 61 months. Data were available for 2 patients before their treatment commenced and 1 patient was untreated. Tasks included reflexive saccades, antisaccades and self-paced saccades, with eye movements recorded by an infrared reflectance eye tracker. Parameters analysed were reflexive saccade gain and latency, asymptotic peak saccadic velocity, HSEM-α (the slope of the peak duration-amplitude regression line), antisaccade error percentage, self-paced saccade count and time between refixations on the self-paced task. Data were analysed by plotting the change from baseline as a proportion of the baseline value at each test time and, where multiple data values were available at each session, by linear mixed effects (LME) analysis.

**Results:**

Examination of change plots suggested some modest sustained improvement in gain, no consistent changes in asymptotic peak velocity or HSEM-α, deterioration in the already poor antisaccade error rate and sustained improvement in self-paced saccade rate. LME analysis showed statistically significant improvement in gain and the interval between self-paced saccades, with differences over time between treated and untreated patients.

**Conclusions:**

Both qualitative examination of change scores and statistical evaluation with LME analysis support the idea that some saccadic parameters are robust indicators of efficacy, and that the variability observed across measures may indicate locally different effects of neurodegeneration and of drug actions.

## Background

Niemann-Pick Type C disease (NPC) is an autosomal recessive neurovisceral disorder of glycosphingolipid metabolism arising from mutations in the NPC1 or NPC2 genes [1, 2]. While no symptom in isolation is pathognomonic for NPC, the presence of a vertical supranuclear gaze palsy (VSGP), particularly of saccadic eye movements, is strongly indicative of NPC when seen in the presence of splenomegaly, ataxia, psychosis and/or cognitive decline [3]. In late-onset patients whose presenting symptom may be schizophrenia-like psychosis or ataxia associated with cognitive decline, evaluation of the range of gaze with smooth eye movements rather than by eliciting saccades can delay proper diagnosis for years [2].

The high prevalence of VSGP in NPC would seem to make it a useful marker for assessing the efficacy of treatment, particularly as ocular motor pathways are relatively well understood. However, by the time diagnosis is confirmed, vertical saccades are often virtually absent. While essentially normal in the earlier stages of the disease [4, 5], definite abnormalities can be identified in horizontal saccadic function as the disease progresses. Their state of being impaired, but not absent, led to the use of the slope of the peak duration versus amplitude regression line, termed horizontal saccadic eye movement alpha (HSEM-α) [6] as one of the principal outcome measures in the clinical trial of miglustat, the first medication approved in a range of jurisdictions for the treatment of NPC [7, 8]. Peak duration is defined as the amplitude of a saccade divided by its peak velocity and HSEM-α corresponds to the inverse of the asymptote Vmax of an exponential curve fitted directly to peak velocity versus amplitude plots [6], sometimes termed the “main sequence.” Two reports arising from this clinical trial have reported that HSEM-α and other improvements seen; e.g., ambulation and swallowing, at 12 months were maintained at 24 months [7, 9]. This implies that neurons responsible for generating the burst of innervation which determines peak saccadic velocity—excitatory burst neurons in the paramedian pontine reticular formation (PPRF)—benefitted from miglustat treatment.

However, peak saccadic velocity is far from the only saccadic parameter which can be measured and whose neural correlates are known. For saccades made in response to the sudden appearance of a target, saccadic accuracy or gain (saccade amplitude divided by stimulus displacement) depends upon the integrity of both the parietal eye fields [10] and cerebellar vermis [11]. Saccadic reaction time or latency can readily be measured for these saccades as well. This is often prolonged in neurodegenerative disorders such as Alzheimer’s disease [12, 13] but less so in progressive supranuclear palsy [14] or frontotemporal dementia [15]. We previously reported latency as intact in NPC [16].

In addition to reflexive or prosaccades made to suddenly appearing stimuli, saccades can also be made under volitional control to consciously chosen locations. One can also consciously suppress saccades which otherwise would be elicited by stimulus appearance (as on the antisaccade task). Saccadic tasks which draw upon these capabilities can be used to assess elements of cognition and the integrity of areas of frontal cortex and projections from there via the basal ganglia to the brainstem. Since NPC has been associated with atrophy of the frontal lobes [17], assessment of frontally mediated saccade tasks could provide additional information about NPC-related declines in these “higher-order” functions and about the integrity of their underlying neural substrates. We recently reported on the performance of a group of nine adult NPC patients and ten age-matched controls on both reflexive and volitional saccade tasks [16]. Several of the parameters (antisaccade error rate, self-paced saccade generation, reflexive saccade gain) were better discriminators between NPC and control groups than HSEM-α. What was not examined in this initial study was whether these measures would show improvement with miglustat treatment similar to that seen in HSEM-α and whether any treatment effect would be sustained. This is the subject of the present report.

## Methods

Nine patients (5 male, 4 female, mean age 32.4 ± 9.71 years) recruited from the Royal Melbourne Hospital were assessed between 2006 and 2013. Intervals between assessments varied between 7 and 12 months. In addition to the recent baseline study [16] some other findings in these patients have been reported elsewhere [18, 19]. All patients provided written informed consent and the study was approved by the Melbourne Health human research and ethics committee (HREC 2005.198). Diagnosis was confirmed with biochemical analysis of cultured fibroblasts, using cholesterol esterification rate and percentage of cells staining abnormally for perinuclear cholesterol. In all but one patient, diagnosis was also confirmed with mutations on the NPC1 gene All patients received 200 mg tds of miglustat except for #9, who received 100 mg tds and #5, who was untreated throughout. Patients #1 and #2 both were recorded twice before the onset of medication. Iturriaga disease severity scale ratings for the patients at their entry to the study are found in Table 1.

**Table 1:** Iturriaga severity rating for patients at the time of their entry into the study

Patient	Illness scale at baseline
1	5
2	12
3	7
4	5
5	11
6	9
7	6
8	5
9	4

As in our earlier studies [16, 18], horizontal eye movements were recorded with a Microguide 1000 infrared limbus tracker and digitised along with a target position signal at 1000 Hz. The system is linear to at least 15 deg and has 0.1 deg sensitivity. Targets were green LEDs on an arc positioned 1.6 m from the subject. Task order was fixed as given below, to ensure that the task most dependent on good calibration and alertness was run first. Tasks used and the parameters assessed for each are as follows: *Prosaccades*: Targets jumped pseudo-randomly to the left and right of fixation so that 60 jumps between 5 and 30° were presented. Analyses of each saccade included peak velocity, gain and latency. Saccade peak velocity was plotted against saccade amplitude and fitted via nonlinear regression with the curve V = Vmax(1 – e^-amp/k^), where Vmax is the asymptotic peak velocity and V and amp are the peak velocity and amplitude of each individual saccade. HSEM-α, the slope of the regression line fitted to a plot of peak duration versus amplitude [6], was also calculated. *Antisaccades*: 39 targets were presented pseudo-randomly at ±5 and 10°. Subjects were instructed to not look at the target but instead to a location at the same distance but in the opposite direction. Practice trials with verbal feedback were given until subjects made at least one correct response, to indicate that they understood the task. Analysis was of the percentage of trials on which an error was made. *Self-paced saccades*: Targets at ±10° were illuminated and subjects instructed to look back and forth between them as rapidly as possible Subjects were verbally encouraged to keep moving during the trial, to minimise the effects of fatigue or inattention. The number of saccades completed in 30 s was tallied. For the subsequent linear mixed effects analysis inter-saccadic interval was also calculated, to allow for trial-by-trial data to be used for this analysis.

### Analyses

**Fig. 1:**
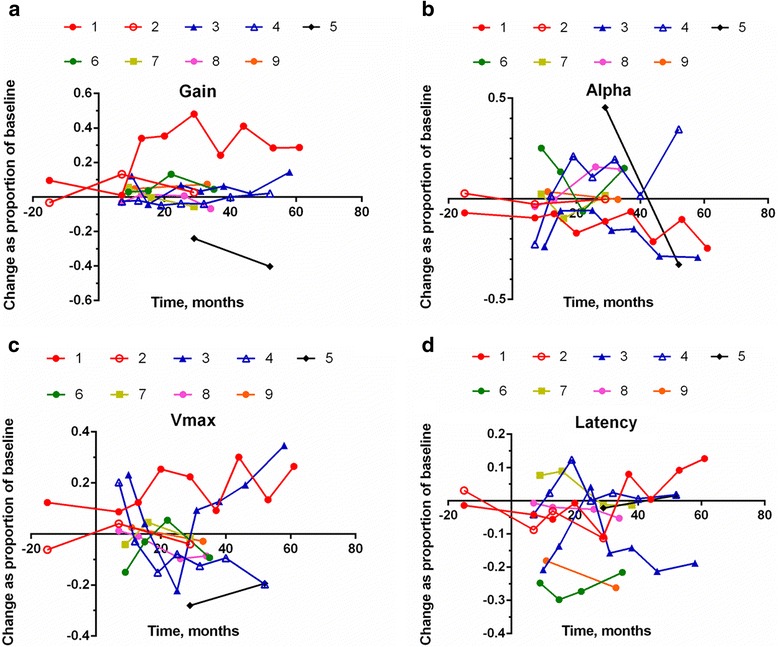
Change scores for (**a**) gain, (**b**) HSEM-α, (**c**) Vmax and (**d**) latency

Data are first shown not in raw form at each time point but as the change from baseline as a proportion of the baseline value. What constitutes improvement or decline depends upon the parameter shown; that is, for self-paced saccade rate, Vmax and gain, an increase from baseline is an improvement and is indicated by data above the horizontal axis. For HSEM-α and antisaccade error rate, a decline in the parameter value is an improvement and is indicated by data below the axis. This was done for the all parameters, as seen in Fig. 1a–d. Patients 1 and 2 were tested before the baseline session; hence, they also have data points before time zero and these sessions and the baseline sessions are represented as untreated data in the linear mixed effects analyses discussed below. The data from 5 and the untreated sessions for 1 and 2 thus constitute the control data in this study.

Quantitative analysis was difficult in a dataset such as this. Patient test-retest intervals varied, not all patients could generate data for all tasks at all times, and the number of repeat visits varied across patients. These limitations precluded the use of repeated measures or mixed analyses of variance. However, an alternative approach which makes maximal use of the data available, linear mixed-effects modelling, was utilised.

Linear mixed-effect (LME) modelling techniques have garnered increasing use, particularly in the field of psycholinguistics (e.g., [20]). While several papers have addressed the advantages of using such a statistical approach over the more traditional analysis of variance (ANOVA) techniques (e.g., [21–23]), there are two main reasons this approach was utilised. First, LME models allow the inclusion of random factors. Here, the number of participants is low, and therefore the LME model considers all of the datapoints for each participant, and controls for the variance between subjects. This gives the analysis more statistical power, and greater sensitivity, than an ANOVA - whereby each participant’s data from each session would have to be condensed to a single cell average. Correspondingly, the second reason for the present use is that LME models can cope well with missing data, as not all participants here have data for the same time points (given that not all sessions were evenly spaced). The models presented here examine the change in factors across time (reported in months), controlling for the baseline variance in performance of our participants. Analysis was conducted using the *lme4* [24] packages in R (version 2.12.0; R Development Core Team, 2010). Data were plotted using the *ggplot* package [25]. We show individual level data from all participants, as well as presenting model data (Coefficient, SE, *t* and *p* values) from separate LME models for treated, untreated (1 patient and 2 patient’s pre-treatment visits) groups, as well as interactions.

## Results

Plots of the change scores are shown in Fig. 1 —as noted above, for gain, Vmax and self-paced saccade rate, positive values are improvements from baseline, while for HSEM-α, latency and antisaccade error rate negative values denote improvement.

For five subjects changes in gain were positive, albeit sometimes by a small amount. Only two subjects showed improvement in Vmax and the related HSEM-α and three subjects’ latency decreased; clearly, however, the data in Fig. 1a to d show significant between-session variability.

**Fig. 2:**
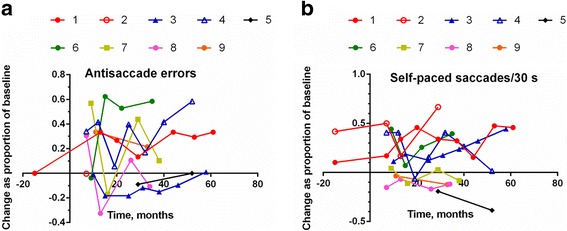
Change scores for (**a**) antisaccade error rate and (**b**) self-paced saccade count

Figure 2a and b show the change values for the two volitional measures.

Antisaccade performance in our patients was poor at baseline and generally became even worse, as evidenced by the large positive change values for most subjects. In contrast, there was a largely sustained improvement in five of the eight treated patients’ self-paced saccade performance; that is, their change scores were positive during their time of treatment, meaning that they were making more self-paced saccades in 30 s. Again, the data vary considerably between sessions.

### LME models

#### Inter-saccadic interval, self-paced saccade task

**Fig. 3:**
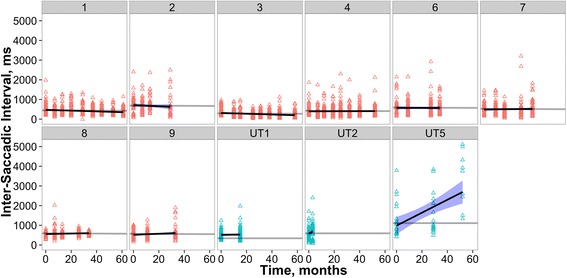
Individual patient ISIs across time, indicated in months since treatment onset. In this and the following two figures, plots with red triangles are treated patients, blue triangles are reported in months since first visit of the untreated patient (UT5) and the two patients recorded before miglustat intervention (UT1 and UT2). The grey lines are the treated and untreated model fits, with intercept varying across participant). The black lines are individually fit regression models with blue shaded ± SE (allowing comparison of individual performance to the overall LME model fit across all participants in that group). Patient numbering is as in Figs. 1 and 2

There was a significant interaction between time and the treatment group on ISI (*p* < .001; Fig. 3). Patients in the treatment group showed a small, but significant decrease in ISI (i.e., a higher rate of self-paced saccade generation) across time (β =–0.001, SE = 0.0003, *t* =–4.334, *p* < .001). In contrast patients in the untreated group showed a significant increase in ISI (i.e., a lower rate of self-paced saccade generation) across time (β = 0.026, SE = 0.004, *t* = 7.258, *p* < .001).

#### Gain

**Fig. 4:**
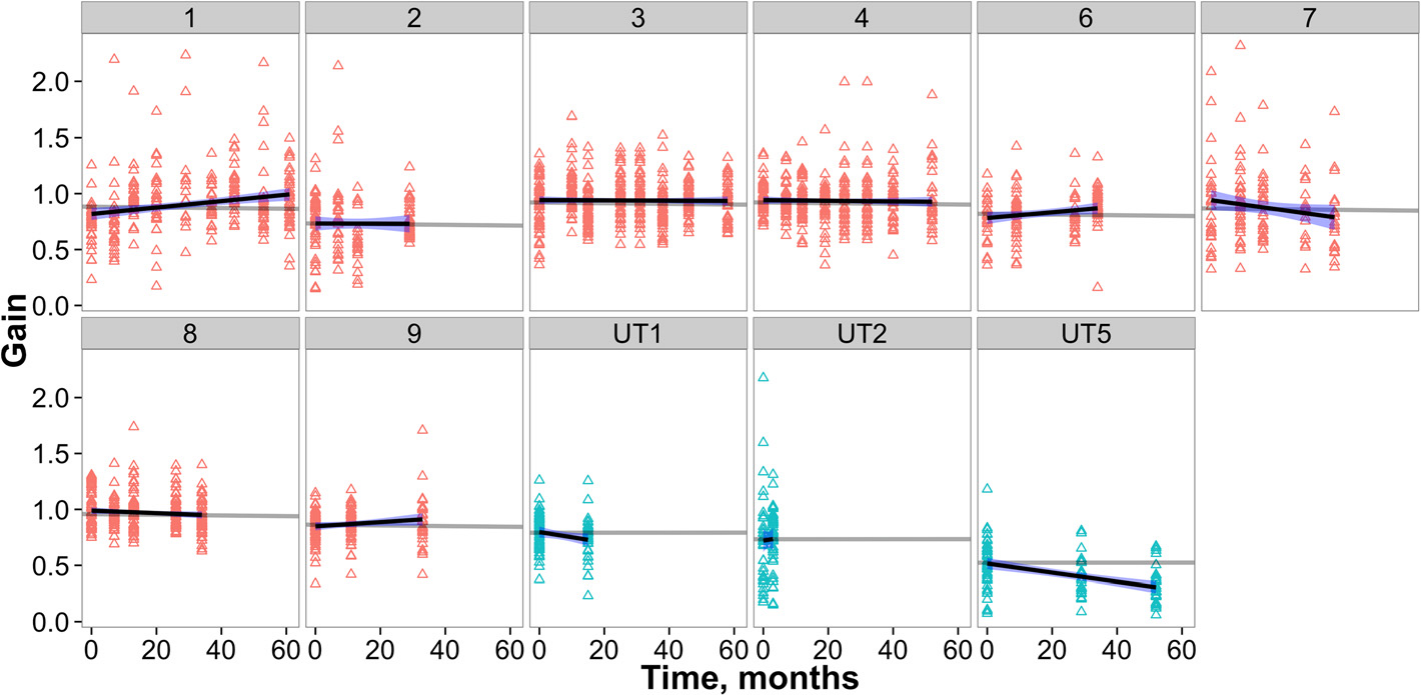
Individual patient gains across time

There was a significant interaction between time and the treatment group on the saccadic gain scores (*p* < .001; Fig. 4). Patients in the treatment group showed a significant increase in gain across time (β = 0.001, SE = 0.0003, *t* = 2.08, *p* = .037). Again, patients in the untreated group showed a different trend with a significant decrease in gain across time (β =–0.004, SE = 0.001, *t* =–4.5, *p* < .001).

#### Latency

**Fig. 5:**
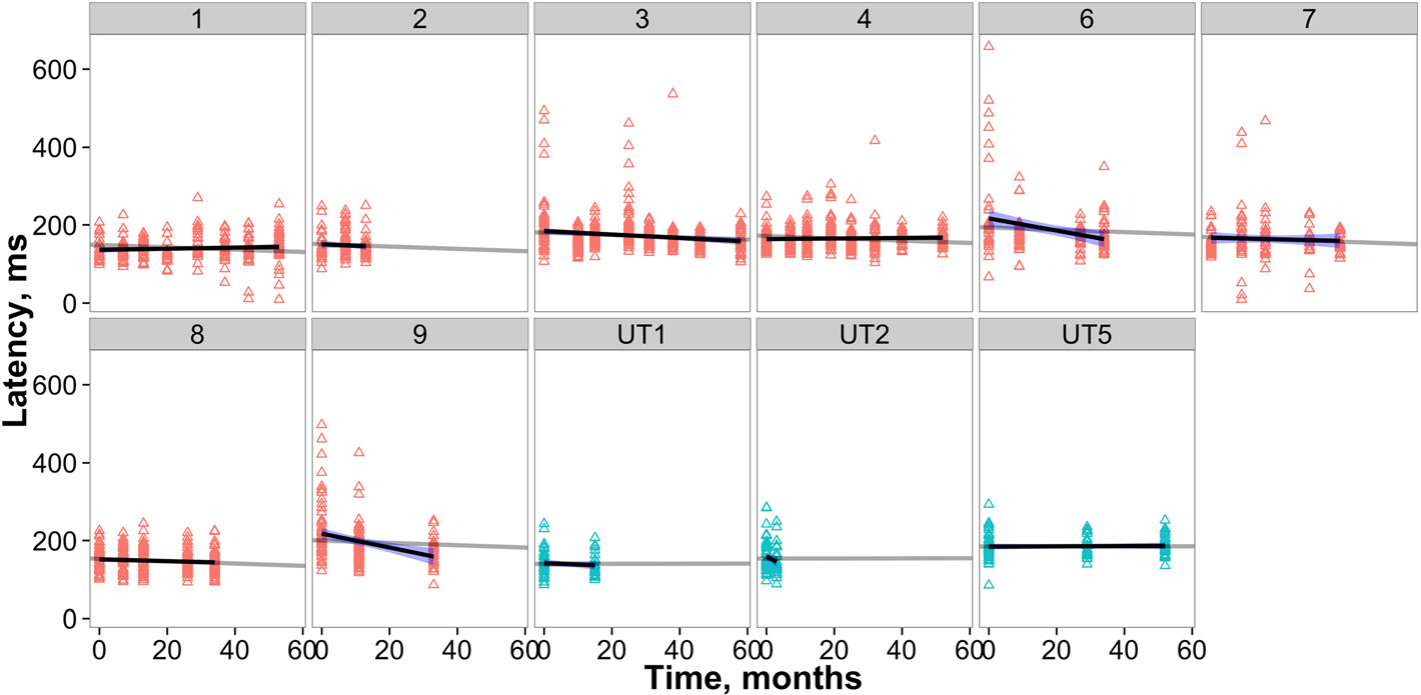
Individual patient latencies across time

There was no significant interaction between time and the treatment group on the saccadic latencies (*p* > .05; Fig. 5). Patients in the treatment group showed a very small significant increase in latency across time (β =–3.010e–04, SE = 7.011e-05, *t* =–4.29, *p* = .04), however, the latency of patients in the untreated group did not change across time (*p* > .05).

**Fig. 6:**
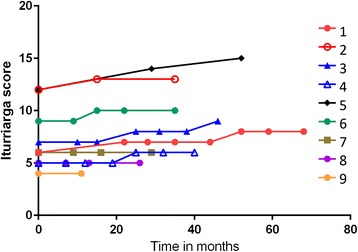
Individual Iturriaga scores across time

We previously reported that baseline disease severity correlated with a number of ocular motor parameters [16]. The values of this assessment over time are shown in Fig. 6.

## Discussion

This report demonstrates the difficulties inherent in conducting a longitudinal treatment study in a rare, progressive and eventually fatal condition. However, even given these limitations it is the most extensive longitudinal study of saccadic eye movement function in NPC to date. The results are broadly consistent with Abel et al. [16], where prosaccade gain, self-paced saccade rate and antisaccade error rate were the best discriminators between patients and controls. In our previous study antisaccade performance was already at close to floor level and in this study we observed that, if anything, it deteriorated further. Since NPC can lead to frontal lobe atrophy [26], presumably the frontal lobe structures essential for reflexive saccade inhibition were already too extensively damaged at baseline to allow for any meaningful treatment response, in the same way that vertical saccades were already essentially absent in our patients upon entry to the study. The marked failure to suppress unwanted reflexive saccades has a behavioural counterpart in the early presentation of disinhibition in the context of other disturbances of executive function [3].

In contrast, it is of interest that self-paced saccades, mediated by the frontal eye fields [27] qualitatively showed sustained improvement in the change plot and this was borne out quantitatively in the LME analysis by showing a significant decrease in intersaccadic interval over time in treated patients and a significant ISI increase in the untreated ones. This task is more readily understood and executed by cognitively impaired patients than is the more complex antisaccade task and its discrimination between controls and NPC patients and its apparent improvement with treatment suggest that it could be a useful addition to future treatment trials.

The measures derived from prosaccades showed less robust results than did self-paced saccades. The parameter used in the miglustat clinical trial [8], HSEM-α, showed improvement in some patients (i.e., lower values) and deterioration in others (i.e., higher values). The same was seen for Vmax, which is effectively the inverse of HSEM-α. Neither of these parameters, being derived from curve-fits to all data collected for a given session, could be quantitatively analysed with LME analysis. The reflexive parameters which could be addressed statistically were gain and latency. The latter measure showed no differences between controls and patients in our baseline study [16] but with LME analysis showed a very small but significant improvement over time. This may have been driven by patients 6 and 9, whose initially abnormally long latencies at baseline (compared to the normal range) normalised in subsequent trials. The preservation of intact horizontal saccadic latency in NPC [16] renders it of little use as a possible marker for treatment response or progression.

Gain, the remaining prosaccade parameter, does show more potential as a treatment marker. It was the only significant prosaccade discriminator between groups in our baseline study and qualitatively in the change plot (Fig. 3a) most data fell above the abscissa, suggesting improvement. This was borne out in the LME analysis, wherein for the NPC group there was a slight but significant improvement in gain over time, while a decrement was seen over time for the untreated subjects.

Clearly this study has a range of limitations: small sample size, different levels of disease severity at intake, the difficulties in maintaining recording quality in a significantly impaired population and variations in test-retest frequency and timing. However, even in this small group we are able to quantitatively demonstrate in some parameters (e.g., self-paced saccade generation and prosaccade gain) an overall improvement with treatment using LME modelling. The LME models were sensitive enough to detect small improvements in these parameters, and differentiate between treated and untreated patients, particularly for ISI (see Fig. 3) where a clear increase was seen in patients during untreated phases compared to a relatively stable (and a slight decrease) in ISI was observed in patients treated with miglustat. Other measures could only be evaluated qualitatively and either appeared to show no strong trend towards either improvement or decline; performance on the antisaccade task, appeared to deteriorate even further from the initially poor baseline.

## Conclusions

As new treatments are proposed and simpler diagnostics become available for NPC, inclusion of measures such as these may be useful objective, quantitative indicators of treatment efficacy in a number of widely divergent brain regions. Indeed, if diagnosis became possible earlier in the disease course, even measures such as vertical saccade performance or antisaccade error rate could be assessed before the loss of their neural substrates rendered them insensitive. In our cohort of adult-onset NPC patients, the changes seen in these frontal and brainstem measures of ocular-motor function support the assertion that miglustat treatment for NPC does have a positive effect on multiple regions in the CNS.

## References

[1] Sevin M, Lesca G, Baumann N, Millat G, Lyon-Caen O, Vanier MT (2007). The adult form of Niemann–Pick disease type C. Brain.

[2] Vanier MT (2010). Niemann-Pick disease type C. Orphanet J. Rare Dis..

[3] Mengel E, Klünemann H-H, Lourenço CM, Hendriksz CJ, Sedel F, Walterfang M (2013). Niemann-Pick disease type C symptomatology: an expert-based clinical description. Orphanet J. Rare Dis..

[4] Rottach KG, von Maydell RD, Das VE, Zivotofsky AZ, Discenna AO, Gordon JL (1997). Evidence for independent feedback control of horizontal and vertical saccades from Niemann-Pick type C disease. Vis Res.

[5] Solomon D, Winkelman C, Zee DS, Gray L, Buettner-Ennever JA (2005). Niemann-Pick Type C disease in two affected sisters: Ocular motor recordings and brain-stem neuropathology. Ann N Y Acad Sci.

[6] Inchingolo P, Spanio M (1985). On the identification and analysis of saccadic eye movements--A quantitative study of the processing procedures. IEEE Trans Biomed Eng.

[7] Patterson MC, Vecchio D, Jacklin E, Abel L, Chadha-Boreham H, Luzy C (2010). Long-term miglustat therapy in children with Niemann-Pick Disease Type C. J Child Neurol.

[8] Patterson MC, Vecchio D, Prady H, Abel L, Wraith JE (2007). Miglustat for treatment of Niemann-Pick C disease: a randomised controlled study. Lancet Neurol.

[9] Wraith JE, Vecchio D, Jacklin E, Abel L, Chadha-Boreham H, Luz C (2010). Miglustat in adult and juvenile patients with Niemann–Pick disease type C: Long-term data from a clinical trial. Molec Genet Metab.

[10] Gaymard B, Ploner CJ, Rivaud S, Vermersch AI, Pierrot-Deseilligny C (1998). Cortical control of saccades. Exp Brain Res.

[11] Ettinger U, Kumari V, Chitnis XA, Corr PJ, Sumich AL, Rabe-Hesketh S (2002). Relationship between brain structure and saccadic eye movements in healthy humans. Neurosci Lett.

[12] Fletcher WA, Sharpe JA (1986). Saccadic eye movement dysfunction in Alzheimer’s disease. Ann Neurol.

[13] Hershey LA, Whicker L, Abel LA, Dell’Osso LF, Traccis S, Grossniklaus D (1983). Saccadic latency measurements in dementia. Arch Neurol.

[14] Pierrot-Deseilligny C, Rivaud S, Pillon B, Fournier E, Agid Y (1989). Lateral visually-guided saccades in progressive supranuclear palsy. Brain.

[15] Garbutt S, Matlin A, Hellmuth J, Schenk AK, Johnson JK, Rosen H (2008). Oculomotor function in frontotemporal lobar degeneration, related disorders and Alzheimer’s disease. Brain.

[16] Abel LA, Bowman EA, Velakoulis D, Fahey MC, Desmond P, Macfarlane MD et al. Saccadic Eye Movement Characteristics in Adult Niemann-Pick Type C Disease: Relationships with Disease Severity and Brain Structural Measures. Plos One. 2012;7(11). doi:10.1371/journal.pone.0050947. 10.1371/journal.pone.0050947 PMC3511378 23226429

[17] Klunemann HH, Elleder M, Kaminski WE, Snow K, Peyser JM, O’Brien JF (2002). Frontal lobe atrophy due to a mutation in the cholesterol binding protein HE1/NPC2. Ann Neurol.

[18] Abel LA, Walterfang M, Fietz M, Bowman EA, Velakoulis D (2009). Saccades in adult Niemann-Pick disease type C reflect frontal, brainstem, and biochemical deficits. Neurol.

[19] Walterfang M, Macfarlane MD, Looi JCL, Abel LA, Bowman EA, Fahey MC (2012). Pontine-to-midbrain ratio indexes ocular-motor function and illness stage in adult Niemann–Pick disease type C. Eur J Neurol.

[20] Baayen RH (2008). Analyzing Linguistic Data: A practical introduction to statistics using R.

[21] Kliegl R, Wei P, Dambacher M, Yan M, Zhou X. Experimental effects and individual differences in linear mixed models: estimating the relationship between spatial, object, and attraction effects in visual attention. Front. Psychol. Quantitative Psychol. Meas. 2011;1. doi:10.3389/fpsyg.2010.00238. 10.3389/fpsyg.2010.00238 PMC3153842 21833292

[22] Baayen RH, Davidson DJ, Bates DM (2008). Mixed-effects modeling with crossed random effects for subjects and items. J Mem Lang.

[23] Kliegl R, Masson MEJ, Richter EM (2010). A linear mixed model analysis of masked repetition priming. Vis Cogn.

[24] Bates D, Maechler M, Bolker B. Linear mixed-effect models using S4 classes. R package version 0.999999-0. 2012. http://CRAN.R-project.org/package=lme4. Accessed 29 June 2015.

[25] Wickham H (2009). ggplot2: elegant graphics for data analysis.

[26] Walterfang M, Fietz M, Fahey M, Sullivan D, Leane P, Lubman DI (2006). The neuropsychiatry of Niemann-Pick Type C disease in adulthood. J. Neuropsychiatry Clin. Neurosci..

[27] Lobel E, Kahane P, Leonards U, Grosbras M-H, Lehericy S, Le Bihan D (2001). Localization of human frontal eye fields: anatomical and functional findings of functional magnetic resonance imaging and intracerebral electrical stimulation. J Neurosurg.

